# Essential Role of Rapid Diagnosis in Subcutaneous Emphysema Following Dental Procedures

**DOI:** 10.7759/cureus.93298

**Published:** 2025-09-26

**Authors:** Nicholas J Carter

**Affiliations:** 1 Department of Emergency Medicine, Evans Army Community Hospital, Fort Carson, USA

**Keywords:** dental procedure complications, head and neck ct, iatrogenic subcutaneous emphysema, post op pneumomediastinum, soft tissue crepitus, subcutaneous emphysema management, tension pneumomediastinum

## Abstract

Subcutaneous emphysema is a rare dental postprocedural complication that can lead to numerous life-threatening conditions. It is important for medical providers to be aware of this complication for early diagnosis and management, which improves patient outcomes. This case report illustrates the clinical course and management decisions in a 35-year-old healthy male who experienced iatrogenic subcutaneous emphysema along with pneumomediastinum following a molar restoration. The considerations of differential diagnosis, treatment, and management are discussed to aid in the medical decision process of the clinician.

## Introduction

Subcutaneous emphysema manifests through numerous different etiologies, including infectious, traumatic, spontaneous, or iatrogenic. Dental procedures are a known cause of the development of cervical facial subcutaneous emphysema and typical advancement to pneumomediastinum of varying degree [1]. Although rare, iatrogenic subcutaneous emphysema is a serious medical condition. The specific incidence rate of iatrogenic subcutaneous emphysema following dental procedures remains unknown. However, it is noted that the use of pressurized, pneumatic dental instruments increases the risk of the introduction of iatrogenic subcutaneous air. Subcutaneous air typically tracks along myofascial planes and can cause life-threatening conditions, including air emboli or clinically significant tracheal compression [2].

## Case presentation

**Figure 1:**
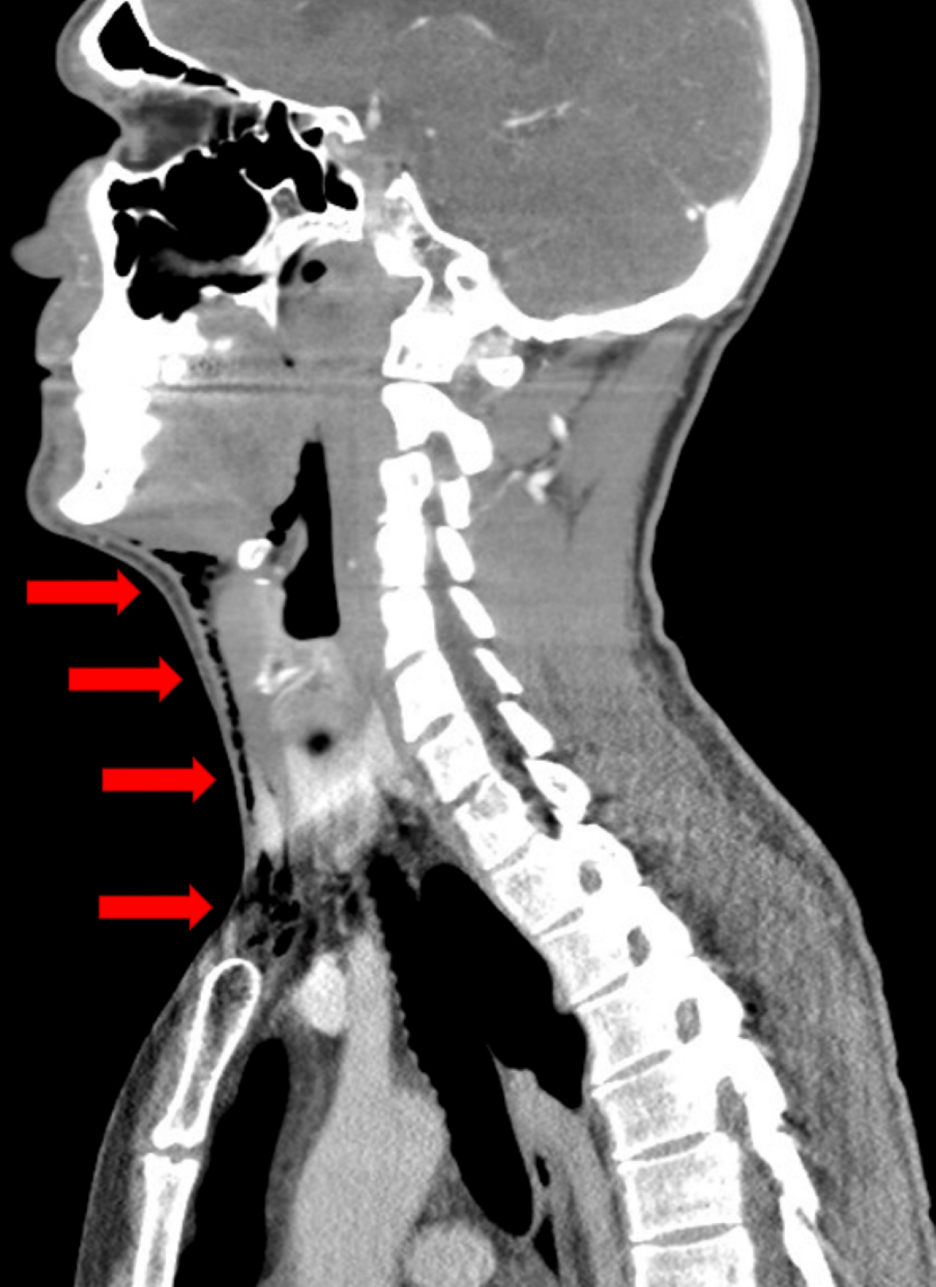
Computed Tomography: Sagittal View Red arrows indicate areas of subcutaneous emphysema.

**Figure 2:**
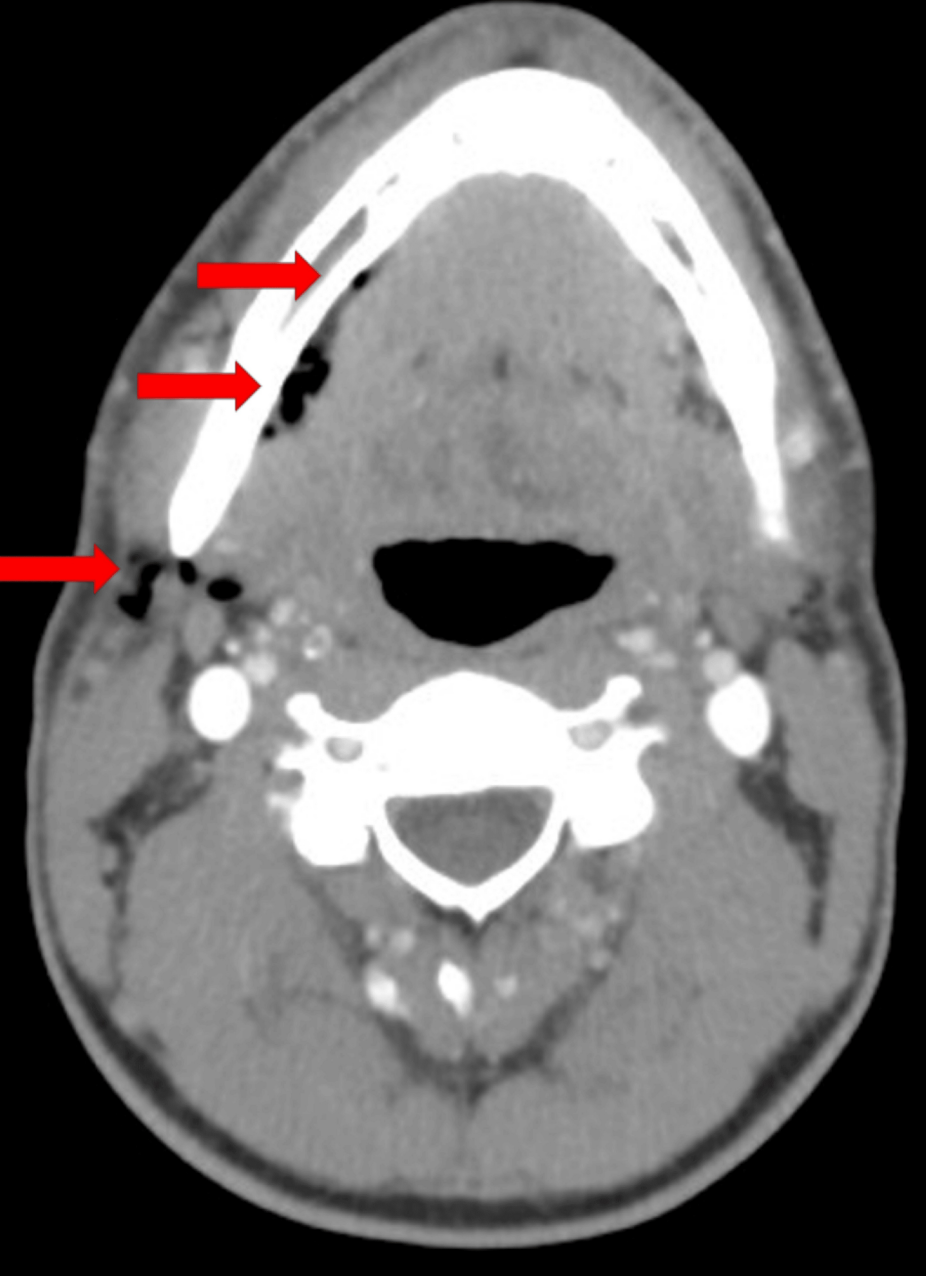
Computed Tomography: Axial/Transverse View Red arrows indicate areas of subcutaneous emphysema.

**Figure 3:**
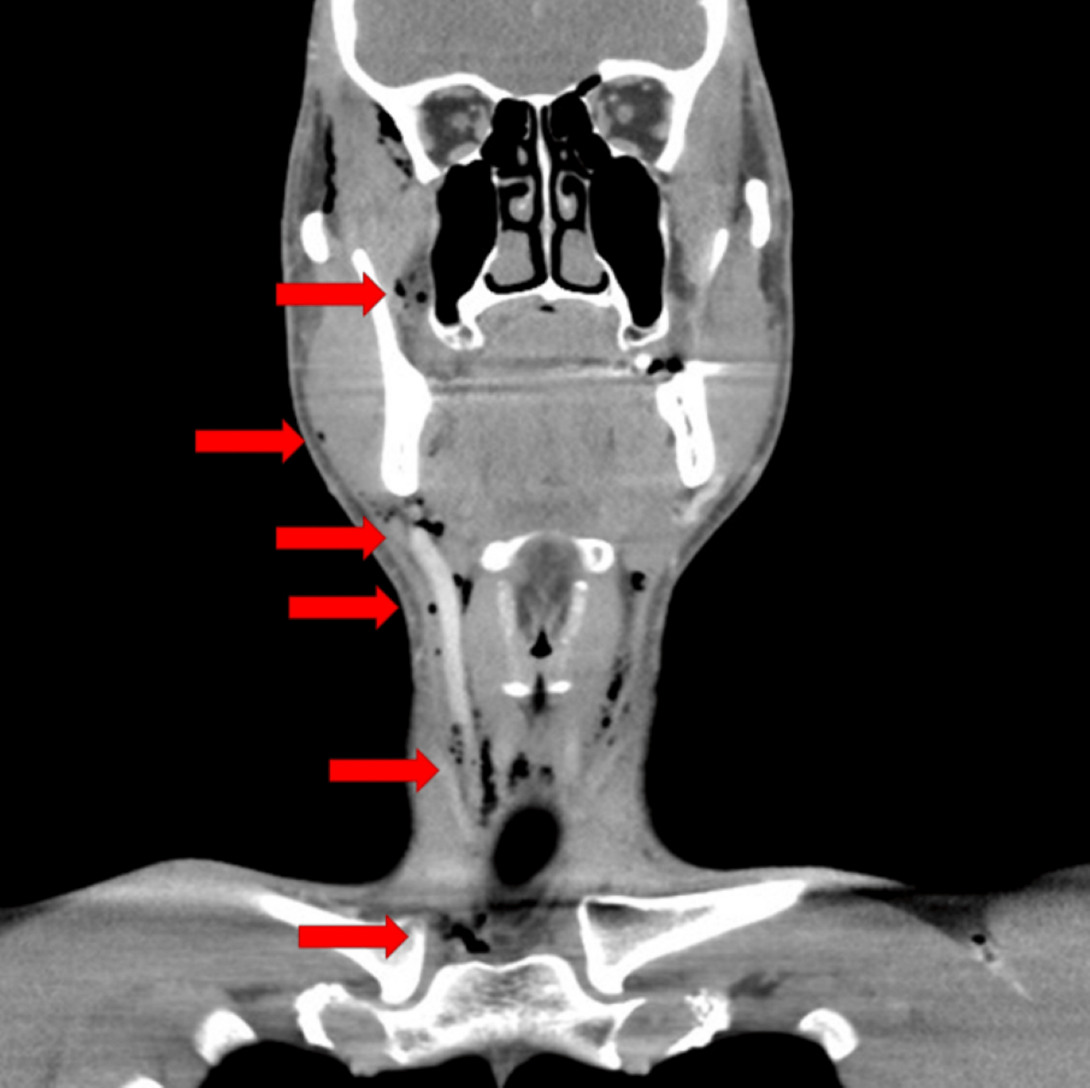
Computed Tomography: Coronal View Red arrows indicate areas of subcutaneous emphysema.

A healthy 35-year-old male began to experience right facial pain. The patient stated his pain worsened over the past 24 hours. The patient reported associated lightheadedness. The combination of lightheadedness and worsening pain led to his evaluation at the emergency department. The patient denied fever, dyspnea, cough, emesis, or trauma. Upon examination, swelling and tenderness were noted over the right mandible. There were no superficial skin changes. Inspection of the mouth revealed erythema and edema of the posterior molar in the right mandible. Additionally, crepitus was palpated anterior to the right sternocleidomastoid muscle, just lateral to the trachea. Further questioning revealed the patient underwent a dental procedure for restoration of the right lower molar two days prior, when two dental fillings were placed in tooth #30. Laboratory evaluation revealed no evidence of leukocytosis, neutrophilia, or elevated acute inflammatory enzymes. Chest radiography was conducted and found to be unremarkable. A computed tomography (CT) scan of the neck with contrast revealed extensive subcutaneous gas throughout the neck and face, particularly right-sided. There was an observable extension of the subcutaneous emphysema, located just above the manubrium, between the clavicles and superior to the proximal clavicles, inferior to the geniohyoid muscles, with extension in the subcutaneous soft tissues of the anterior neck (Figure 1). There was more mild subcutaneous emphysema noted along the medioinferior right mandible, inferior to the mylohyoid ridge (Figure 2). Finally, more subcutaneous gas was noted to be tracking superiorly in the right face, lateral to the masseter and circumferentially around the temporal muscle (Figure 3). There was no evidence of inflammation, fat stranding, fluid collection, or abscess. A dental and oral maxillary facial surgeon evaluated the patient. The patient was admitted to the intensive care unit (ICU) due to concern for tension phenomenon given proximity to the airway and major vasculature. The patient declined a surgical procedure to evacuate subcutaneous air. As such, he was observed while allowing the gas to absorb into soft tissues. Over the next 24 hours, the patient's symptoms markedly improved without specified intervention. The patient was prescribed Augmentin prophylactically, though suspicion for bacterial etiology was exceedingly low given the clinical picture, timing to dental procedure, and no evidence of systemic inflammation. Overall, the patient's subcutaneous emphysema was likely introduced iatrogenically during the dental procedure. He was discharged in good condition with close follow-up. He was instructed to complete his antibiotic course with close follow-up. His subcutaneous emphysema was 90% improved by post-operative day 4 with no indication for directed follow-up. No further complications were reported, and the patient's symptoms have resolved. He has returned to his baseline health.

## Discussion

This case highlights the importance of early evaluation, recognition, and diagnosis of dental procedure complications, particularly subcutaneous emphysema, due to the development of life-threatening conditions and dissemination of bacteria [3] leading to necrotizing fasciitis; both etiologies can cause subcutaneous emphysema and lead to death. Additionally, the differential diagnosis should include, but not be limited to, angioedema, hematoma, hypersensitivity reaction [4], mechanical ventilation, intubation, esophageal perforation, barotrauma, spontaneous pulmonary injury, and penetrating trauma. The diagnosis of subcutaneous emphysema is suspected when palpating subdermal crepitus and confirmed with radiography, including plain films or CT [5]. The use of prophylactic antibiotics is recommended regardless of laboratory results, as the presence of subcutaneous emphysema represents a compromise of tissues that naturally communicate with air, as in this case, or infection containing bacteria; it is recommended that clinicians should not wait for systemic signs and symptoms prior to initiation of antibiotic treatment. Any case involving a pneumomediastinum or pneumothorax requires hospital admission and airway monitoring with surgical intervention in a decompensating patient [6]. The decision to admit the patient to the ICU versus a general inpatient ward should be based on the clinical stability of the patient as well as the timing of reevaluation. For instance, cases requiring hourly or less vital sign monitoring should be admitted to the ICU or ICU “step-down” unit due to limitations in nursing staff-to-patient ratios. If subcutaneous air evacuation is not indicated or declined by the patient, the air is suspected to absorb and resolve within 10 days outside of severe or complicating circumstances [7].

## Conclusions

Early recognition of dental postprocedural complications is vital to identify life-threatening sequela, including pneumomediastinum, air emboli, necrotizing fasciitis, and clinically significant compression of tracheal or neurovascular structures. Therefore, this case report aims to increase clinician awareness of dental postprocedural subcutaneous emphysema along with the considerations of management. Further, as this case highlights, a surgical intervention is not always required and depends on the patient's clinical status.
